# Assessment of Iron Deposition and White Matter Maturation in Infant Brains by Using Enhanced T2 Star Weighted Angiography (ESWAN): R2* versus Phase Values

**DOI:** 10.1371/journal.pone.0089888

**Published:** 2014-02-25

**Authors:** Ning Ning, Lei Zhang, Jie Gao, Yumiao Zhang, Zhuanqin Ren, Gang Niu, Yongming Dai, Ed X. Wu, Youmin Guo, Jian Yang

**Affiliations:** 1 Radiology Department of The First Affiliated Hospital, Xi’an Jiaotong University, Xi’an, Shaanxi, People’s Republic of China; 2 Radiology Department of Baoji Center Hospital, Baoji, Shaanxi, People’s Republic of China; 3 Laboratory of Biomedical Imaging and Signal Processing, The University of Hong Kong, Hong Kong SAR, People’s Republic of China; 4 Nuclear Medicine Department of The Second Affiliate Hospital, Xi’an Jiaotong University, Xi’an, Shaanxi, People’s Republic of China; Institute of Automation, Chinese Academy of Sciences, China

## Abstract

**Background and Purpose:**

Iron deposition and white matter (WM) maturation are very important for brain development in infants. It has been reported that the R2* and phase values originating from the gradient-echo sequence could both reflect the iron and myelination. The aim of this study was to investigate age-related changes of R2* and phase value, and compare their performances for monitoring iron deposition and WM maturation in infant brains.

**Methods:**

56 infants were examined by enhanced T2 star weighted angiography (ESWAN) and diffusion tensor imaging in the 1.5T MRI system. The R2* and phase values were measured from the deep gray nuclei and WM. Fractional anisotropy (FA) values were measured only in the WM regions. Correlation analyses were performed to explore the relation among the two parameters (R2* and phase values) and postmenstrual age (PMA), previously published iron concentrations as well as FA values.

**Results:**

We found significantly positive correlations between the R2* values and PMA in both of the gray nuclei and WM. Moreover, R2* values had a positive correlation with the iron reference concentrations in the deep gray nuclei and the FA in the WM. However, phase values only had the positive correlation with PMA and FA in the internal capsule, and no significant correlation with PMA and iron content in the deep gray nuclei.

**Conclusions:**

Compared with the phase values, R2* may be a preferable method to estimate the iron deposition and WM maturation in infant brains.

## Introduction

Iron deposition and white matter (WM) maturation in infants are of great importance for the human brain development [1]. In physics, iron and myelin have the magnetic susceptibility, which may change the local magnetic field when exposed to an external magnetic field [2]. In light of this property, the current MR imaging techniques could be used to detect and quantify them in vivo and may provide the potential means to monitor the development of human brain [3]–[8].

Previous studies have shown that the transverse relaxation time (T2) or transverse relaxation rate (R2 = 1/T2) reflects the nuclear interaction of adjacent protons (spins) [2], [9], which could be affected by water content [10]. On the other hand, T2* or R2*(R2* = 1/T2*) reflects the combination of the nuclear interaction and field inhomogeneity caused by the presence of paramagnetic or diamagnetic substances such as iron and myelin [1]. Regarding to the variations of the brain iron concentration in the gray matter, R2* was shown to be more sensitive than R2 [11]. Moreover, R2* was also useful to detect myelination in WM depending on the tissue’s orientation relative to the static magnetic field (B0) [12]. Although the field-dependent R2 increase (FDRI) and R2’ (R2’ = R2*- R2) values would be more specific for iron quantification in the brain, the complexity of acquisition methods and excessively long acquisition time render them to be impractical for infants [5], [10], [13], [14]. Susceptibility weighted imaging based on a high-spatial-resolution three-dimensional gradient-echo sequence utilizes phase information as an extra source of contrast [15]. It reveals the phase shift and enhances the visualization of iron, calcification, veins, as well as the blood by-products according to their paramagnetic or diamagnetic properties [9], [15], [16]. For instance, in a right-handed system, paramagnetic substances such as iron can result in accentuating the magnetic field inhomogeneity and cause a negative phase shift or/and R2* increase relative to the surrounding parenchyma. However, the myelination is characterized by a positive phase shift or/and R2* increase. These features enable the possibility of evaluating iron deposition [7], myelination [8], and susceptibility induced gray-white matter contrast [17].

Quantification of iron and WM development in children, adults, and animals have been reported by using the MR parameters of phase and R2* in several previous studies [1], [7], [8], [10], [13], [18]–[20]. Recently, a few studies focused on the brains of neonates and infants. In the phase imaging, the myelin showed an age-dependent change and indicated as an essential and dynamic source of phase contrast in full-term neonates [21]. Moreover, R2* values in the lenticular nucleus and the posterior limb of internal capsule (PLIC) increased with age and could be used to distinguish the preterm neonates at term-equivalent age and full-term controls [19]. Therefore, the R2* and phase may be the potent parameters to evaluate the early brain maturation process. In this study, the parameters of R2* and phase values calculated from a sequence of enhanced T2 star weighted angiography (ESWAN) were used to observe the age-related iron changes and WM development in the infant brains. The reliability and potentiality for monitoring iron deposition and WM maturation were compared between the phase and R2* during the first year after birth.

## Materials and Methods

### Subjects

The study complied with institutional guidelines and regulations and was approved by the Ethics Committee of the First Hospital of Medical School, Xi’an Jiaotong University. Written informed consent was obtained from the subjects’ parents. In total, 56 infants whose postmenstrual age (PMA) [22] ranged from 37 to 91 weeks (54±14 weeks) were enrolled in this study, including 36 boys (55±14 weeks) and 20 girls (52±15 weeks). There was no significant difference in age distribution between the genders (t = 0.862, *P* = 0.392, independent-samples t test). All the subjects were enrolled according to the inclusion and exclusion criteria. The inclusion criteria were as follows: (1) birth weight appropriate for gestational age [22]; (2) no history of neurological or psychiatric conditions; and (3) clinically asymptomatic at the time of registration. The exclusion criteria were: (1) a history of cerebral infection; (2) clinical evidence of seizures; (3) evidence of asphyxia; (4) hypoxic-ischemic encephalopathy, intracranial hemorrhage or WM damage; (5) metabolic disorder; (6) abnormalities of the mother during the pregnancy such as iron deficiency or diabetes mellitus; or (7) any other abnormalities in T1-weighted images or T2-weighted images.

### MR Imaging

All the MR images were obtained by a 1.5-T system (HD, General Electric Co., Waukesha, Wisconsin, USA) equipped with a commercial 8-channel head coil. The infants were well sedated with 25 mg oral chloral hydrate per kilogram of body weight before imaging. Infant motion was minimized by wrapping them in a vacuum immobilization mat with earplugs and earmuffs to protect hearing. The infants were continuously monitored by an investigator during the scanning.

Sagittal T2-weighted images were acquired with a fast spin-echo sequence to locate the anterior and posterior commissures. ESWAN was performed using a three-dimensional multiple gradient echo sequence with slices paralleling to the anterior–posterior commissural line. Imaging parameters were TR = 88.1 ms, number of echoes = 11, TE = 38∼75 ms, echo interval = 3.7 ms, flip angle = 20°, slice thickness/gap = 3 mm/0 mm, NEX = 1, FOV = 24×24 cm^2^, matrix = 256×256, the number of slices = 24 and a total acquisition time = 217 s. Diffusion tensor imaging (DTI) was performed with the following parameters: 15 gradient directions, b = 1000 s/mm^2^, TR = 5950 ms, TE = 94.7 ms, slice thickness/gap = 4 mm/0 mm, FOV = 24×24 cm^2^, matrix = 128×128, readout bandwidth = 250 kHz, and the number of slices = 18 with a total acquisition time = 210 s. Fractional anisotropy (FA) images were obtained for further analysis.

### Post-processing and Measurement

The post-processing was performed on ADW4.3 workstation (HD, General Electric Co., Waukesha, Wisconsin, USA). The phase, R2* and FA maps were constructed after removing the image distortion by the workstation software automatically. In the corrected phase image, phase values ranged from –π to +π [15], [16]. The R2* map was obtained from the magnitude images of all eleven echoes by using a signal intensity fitting algorithm [1].

Two trained neuroradiologists blinded to the subjects’ information, manually traced the regions of interest (ROIs) and recorded the values independently. The mean values of the two measurements were taken as the representative values for the final analysis.

**Figure 1:**
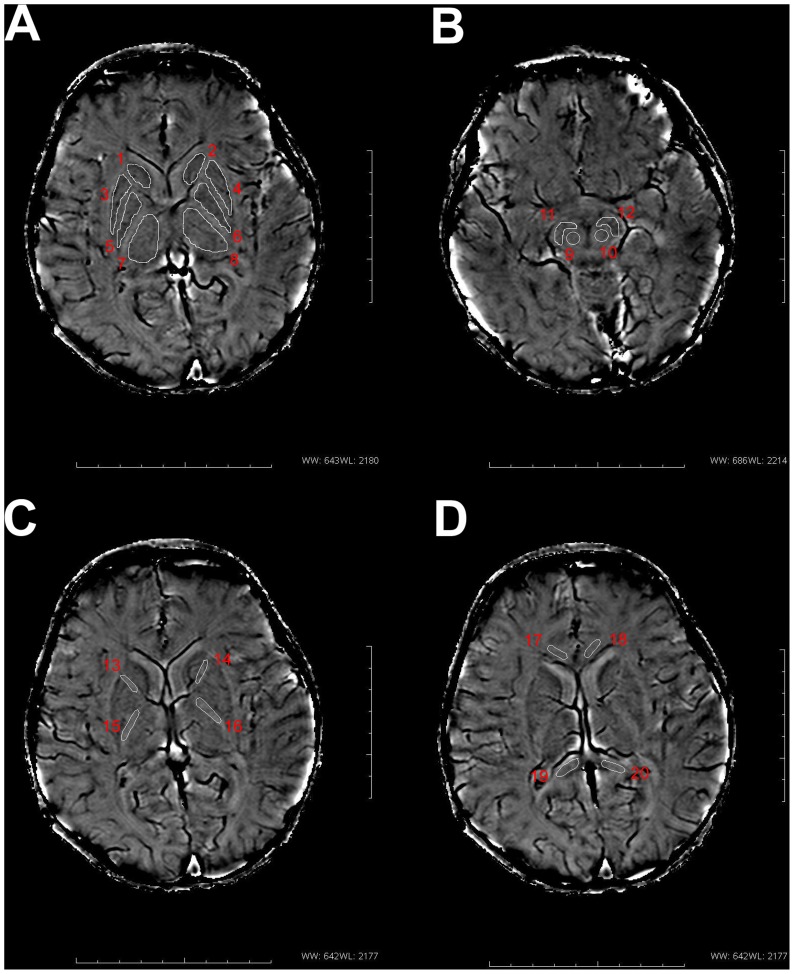
Regions of interest (ROIs) in phase images. ROIs placement is illustrated on images from an infant brain(postmenstrual age = 90 w)(A) 1, 2 : caudate nucleus; 3, 4: putamen; 5, 6: globus pallidus; 7, 8: thalamus. (B) 9, 10: red nucleus; 11, 12: substantia nigra. (C) 13, 14: anterior limb of the internal capsule; 15, 16: posterior limb of the internal capsule. (D) 17, 18: genu of the corpus callosum; 19, 20: splenium of the corpus callosum.

The ROIs were outlined manually based on their anatomical locations and the boundaries shown in the filtered phase images (Figure 1) and then copied to the corresponding R2* maps which guaranteed that the boundaries were exactly same in both images. R2* and phase values of ten anatomical regions were measured bilaterally including: the caudate nucleus (CN), putamen (PUT), globus pallidus (GP), thalamus (THA), red nucleus (RN), substantia nigra (SN), anterior limb of the internal capsule (ALIC), PLIC, genu of the corpus callosum (GCC), and splenium of the corpus callosum (SCC). The criteria of drawing ROIs in phase map was described as follows according to the previous study [7]: (1) One single slice, which showed the largest area and the most well-defined border was selected for each structure and analyzed (Figure 1A–D). As a further condition, slices which were severely affected by artifacts were not used. (2) The bright boundaries outside the structures were also avoided because this large positive phase shift was most likely associated with the iron-induced dipolar field patterns in the tissue. (3) Structures were zoomed by a factor of four to make the boundaries easier to determine and the area of the structure more accurately drawn. The details of drawing ROIs in phase map were as follows. The CN, GP, PUT and the THA were chosen in the same slice with the highest contrast in the basal ganglia region and to avoid the small veins (Figure 1A). The RN and SN were drawn in the same slice which showed the ring-like hyperintensity signal outside the RN, and showed no fusion of them (Figure 1B). Moreover, the ALIC and PLIC were drawn to avoid the deep gray nuclei (Figure 1C). The GCC and RCC in one slice were outlined respectively on both sides to avoid the small veins (Figure 1D). FA values were measured bilaterally only in the above four WM ROIs in the FA maps.

### Statistical Analysis

The inter-observer variations of R2*, phase and FA values were respectively analysed by Bland-Altman analysis. Results were presented as Mean ± SD. Regional iron concentrations in the CN, PUT, and GP were estimated from the empirical equations reported by Hallgren and Sourander [23], as follows, CN: b = 9.66 [1– exp (−0.05 a)] +0.33, PUT: b = 14.62 [1– exp (−0.04 a)] +0.46, GP: b = 21.41 [1– exp (−0.09 a)] +0.37, where a is age and b is the iron concentration.

For brain iron change, the correlation between R2* or phase values and iron concentrations in the CN, PUT and GP were analyzed. For WM change, the correlation between R2* or phase values and FA were analyzed. Graphpad Prism (6.01, Graphpad software Inc. CA, USA) and SPSS for Windows (13.0, SPSS Inc. Chicago, IL) were used to do the statistical analyses and graphics. Statistical differences with *P*<0.05 were considered significant. Correlations were considered to be high, moderate, or poor when correlation coefficients (r) were >0.7, 0.4–0.7, or <0.4, respectively [24].

## Results

### Agreement Analysis

**Figure 2:**
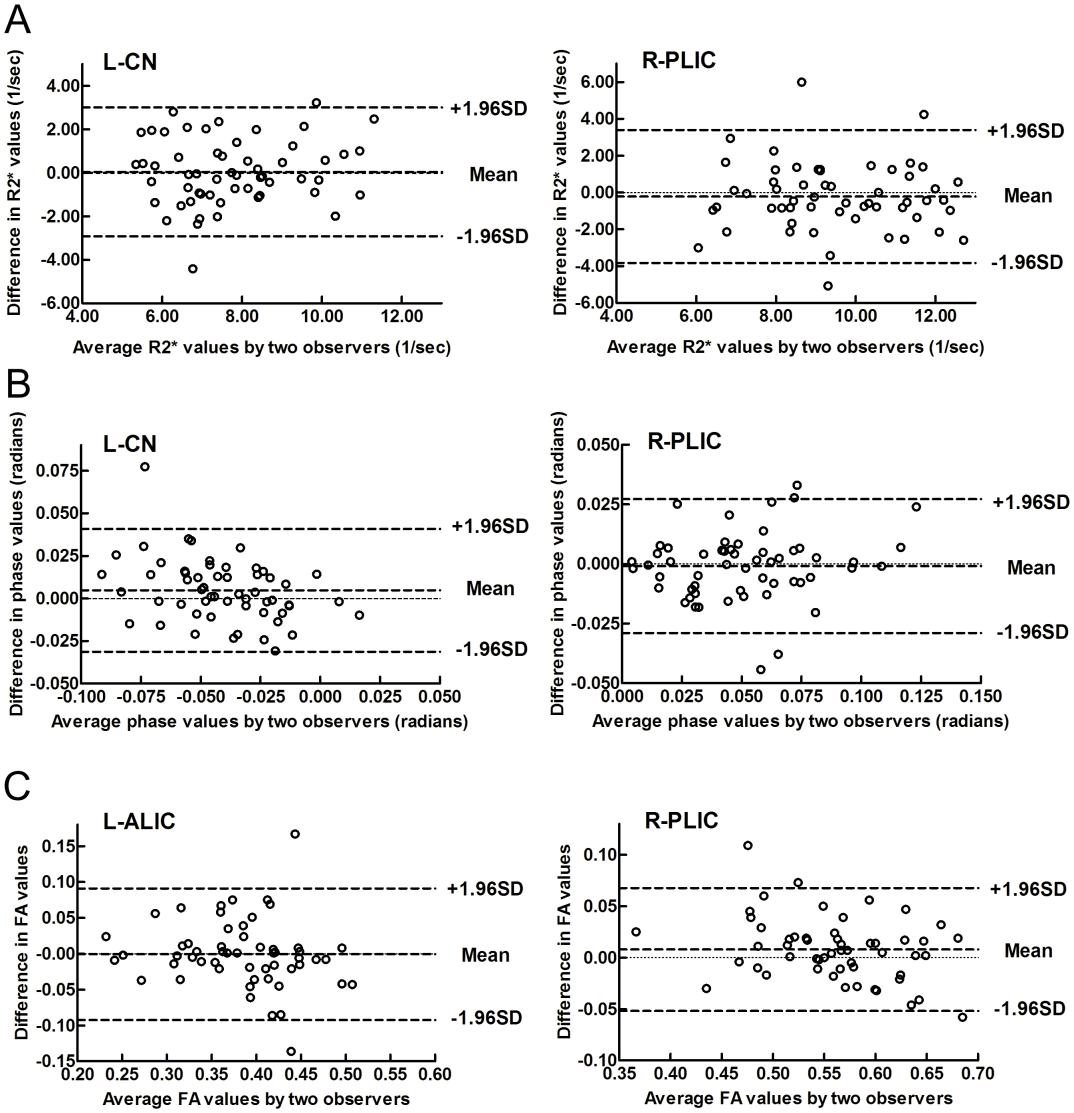
Bland-Altman plots showing inter-observer variability of measurements. (A) Upper: for R2* values, (B) middle: for phase values, and (C) lower: for FA values. CN: caudate nucleus; ALIC: anterior limb of the internal capsule; PLIC: posterior limb of the internal capsule; L: left; R: right.

The inter-observer variations between two trained neuroradiologists in the R2*, phase and FA values have been respectively identified by Bland-Altman analysis. Most of the scatters are located within the limit of agreement (±1.96×SD), and the average difference (Mean) is approximately 0, which indicate a good agreement in two observers for the R2*, phase and FA values in this study (taking the CN, ALIC and PLIC as examples in Figure 2).

### Developmental Changes of R2* and Phase Values in Deep Gray Nuclei

**Figure 3:**
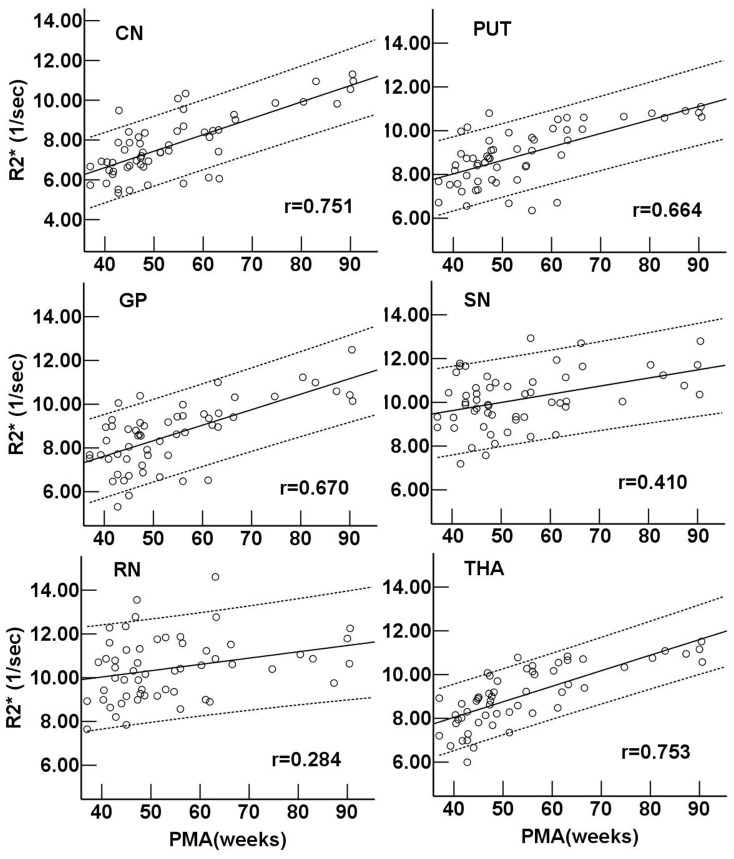
Regional R2* value vs. postmenstrual age in deep gray nuclei. Pearson correlation analysis showed a positive correlation between the R2* values and postmenstrual age in gray nuclei (*P*<0.001). r is the coefficient of correlation. CN: caudate nucleus; PUT: putamen; GP: globus pallidus; THA: thalamus; RN: red nucleus; SN: substantia nigra.

**Figure 4:**
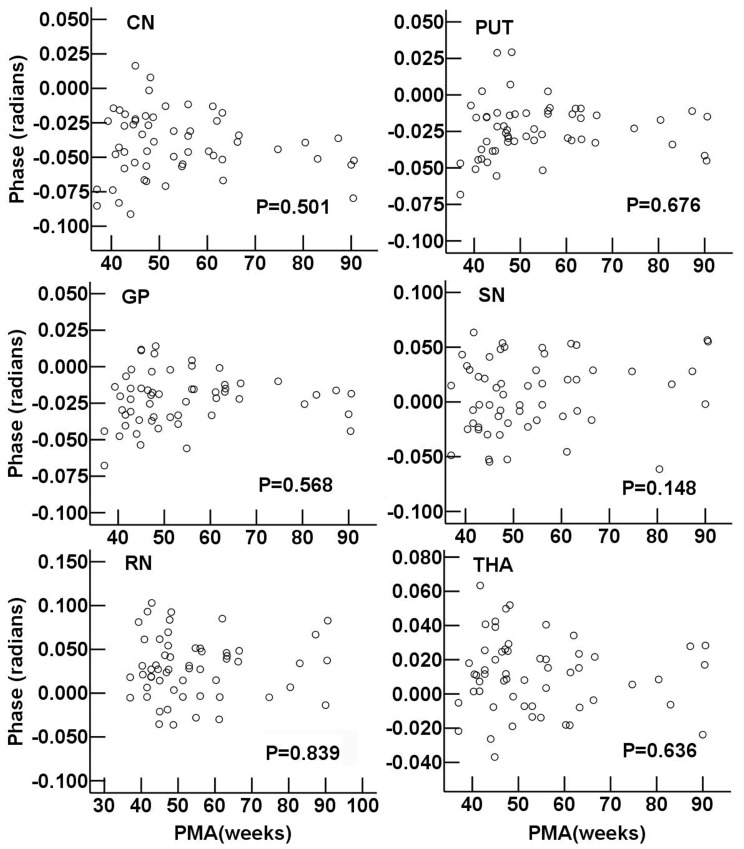
Regional phase value vs. postmenstrual age in deep gray nuclei. Pearson correlation analysis showed no significant correlation between the phase values and postmenstrual age in gray nuclei (*P*>0.05). The *P* values of the linear correlation in different regions are shown. CN: caudate nucleus; PUT: putamen; GP: globus pallidus; THA: thalamus; RN: red nucleus; SN: substantia nigra.

During the infant period, R2* values in the CN and THA showed a highly positive correlation with PMA (r = 0.751 and 0.753, respectively, *P*<0.001). R2* values in the PUT and GP exhibited a moderate correlation with PMA (r = 0.664 and 0.670, respectively, *P*<0.05). Moreover, poor correlations were showen between R2* values with PMA in the RN and SN (r = 0.284 and 0.410, respectively, *P*<0.05) (Figure 3). There was no significant correlation between phase value and PMA in each deep gray nucleus (*P*>0.05) (Figure 4).

**Figure 5:**
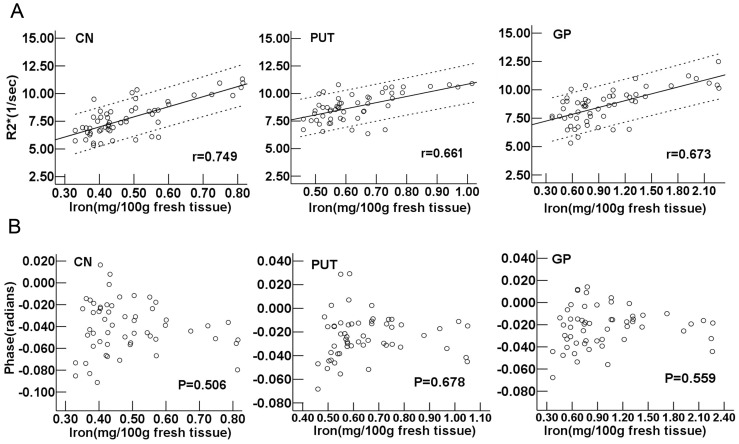
R2* and phase values vs. iron concentration calculated by equations in CN, PUT and GP. (A) Upper: R2* vs. iron concentration, and (B) lower: phase vs. iron concentration. Pearson correlation analysis showed a strongly positive correlation between the R2* values and the iron concentrations (*P*<0.001). r is the coefficient of correlation. As for phase values, no correlations with the iron concentrations were found (*P*>0.05). CN: caudate nucleus; PUT: putamen; GP: globus pallidus.

According to the corrected age [22], the regional iron concentrations in CN, PUT and GP were estimated by using the empirical equations from Hallgren and Sourander’s report [23]. The scatter plots and regression line of R2* corresponding to iron concentration were shown in Figure 5A. The Pearson correlation analysis revealed a highly positive correlation between the R2* values and the iron concentrations in the CN, PUT and GP (r = 0.749, 0.661 and 0.673, respectively, *P*<0.001). For the phase value, no correlation with the iron concentrations was observed (*P*>0.05) (Figure 5B).

### Developmental Changes of R2* and Phase Values in WM

**Figure 6:**
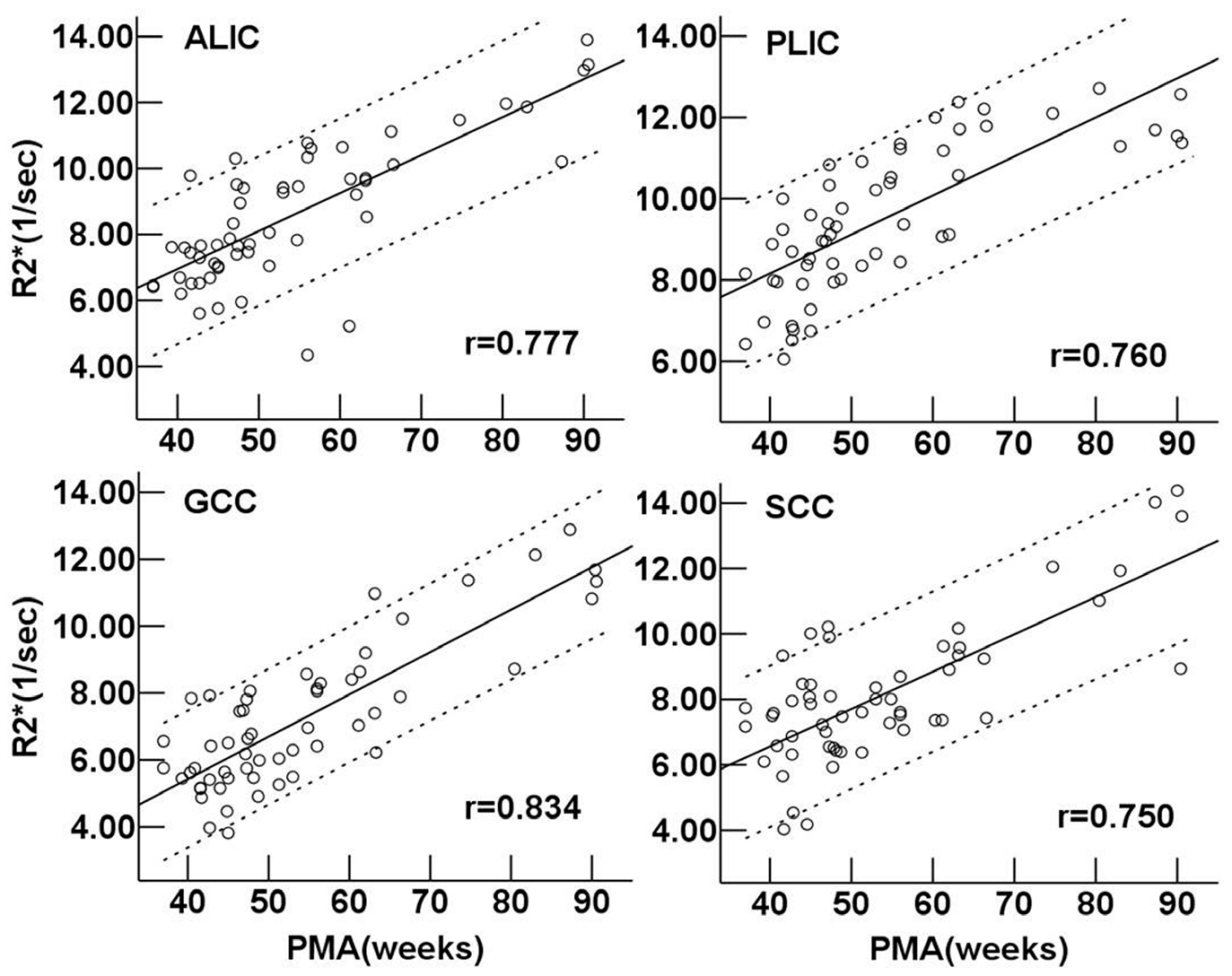
Regional R2* value vs. postmenstrual age in the white matter regions. Pearson correlation analysis showed a positive correlation between the R2* values and postmenstrual age in the white matter regions (*P*<0.001). r is the coefficient of correlation. ALIC: anterior limb of the internal capsule; PLIC: posterior limb of the internal capsule; GCC: genu of the corpus callosum; SCC: splenium of the corpus callosum.

**Figure 7:**
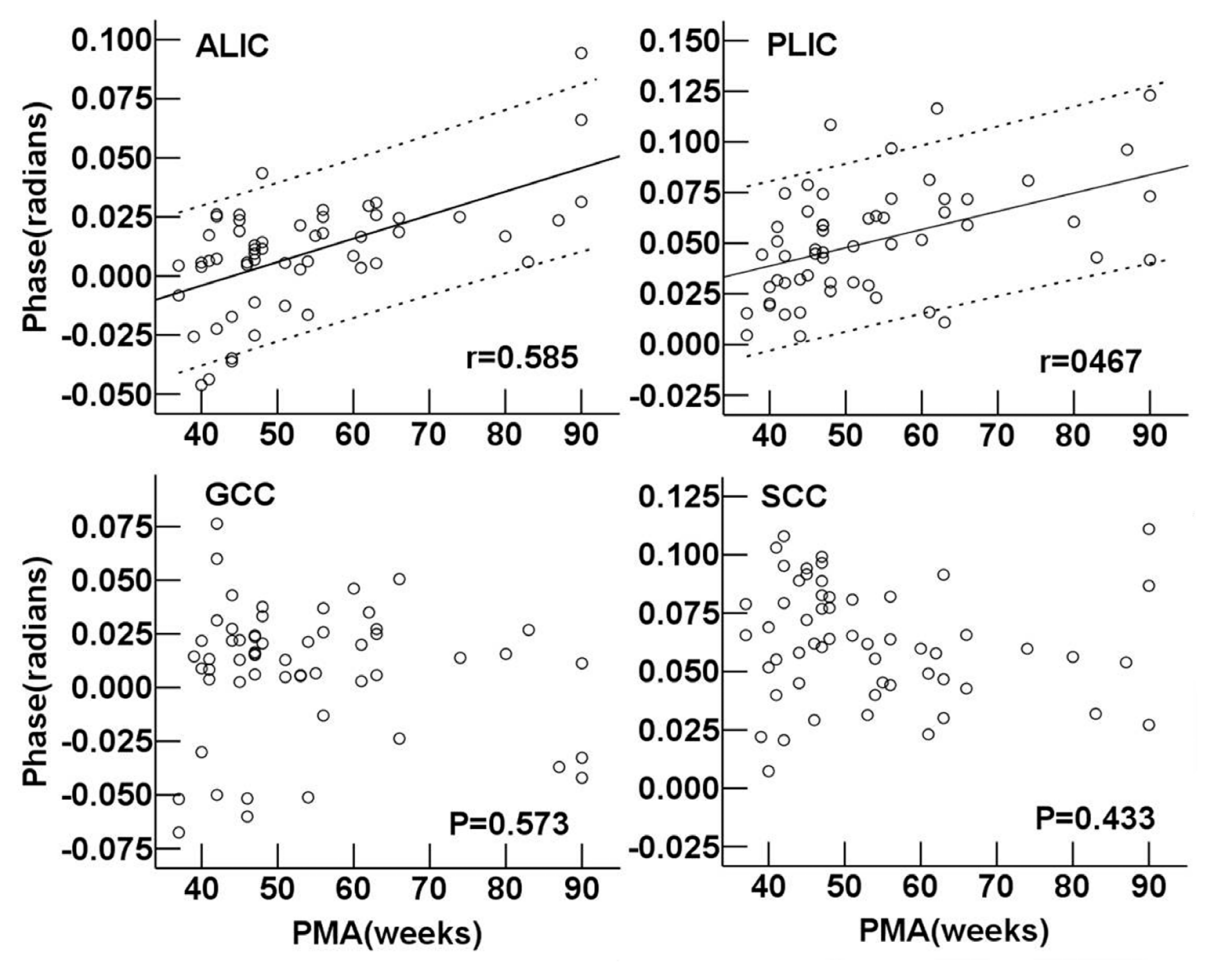
Regional phase value vs. postmenstrual age in the white matter regions. Pearson correlation analysis showed a positive correlation between the phase values and postmenstrual age in ALIC and PLIC (*P*<0.001). r is the coefficient of correlation. As for phase values in GCC and SCC, no correlations with postmenstrual age were found (*P*>0.05). ALIC: anterior limb of the internal capsule; PLIC: posterior limb of the internal capsule; GCC: genu of the corpus callosum; SCC: splenium of the corpus callosum.

As seen in Figure 6, the R2* values of the ALIC, PLIC, GCC and SCC all showed highly positive linear correlation with PMA (r = 0.777, 0.760, 0.834 and 0.750, respectively, *P*<0.001). In neonates, the phase values in the PLIC and SCC were significantly higher than those in the ALIC and GCC respectively (*P = *0.037 and *P = *0.027). The phase values of the ALIC and PLIC showed a moderately positive correlation with PMA (r = 0.585 and 0.467, respectively, *P*<0.001) (Figure 7). However, there was no significant correlation between the phase values and PMA in the GCC and SCC (*P*>0.05).

**Figure 8:**
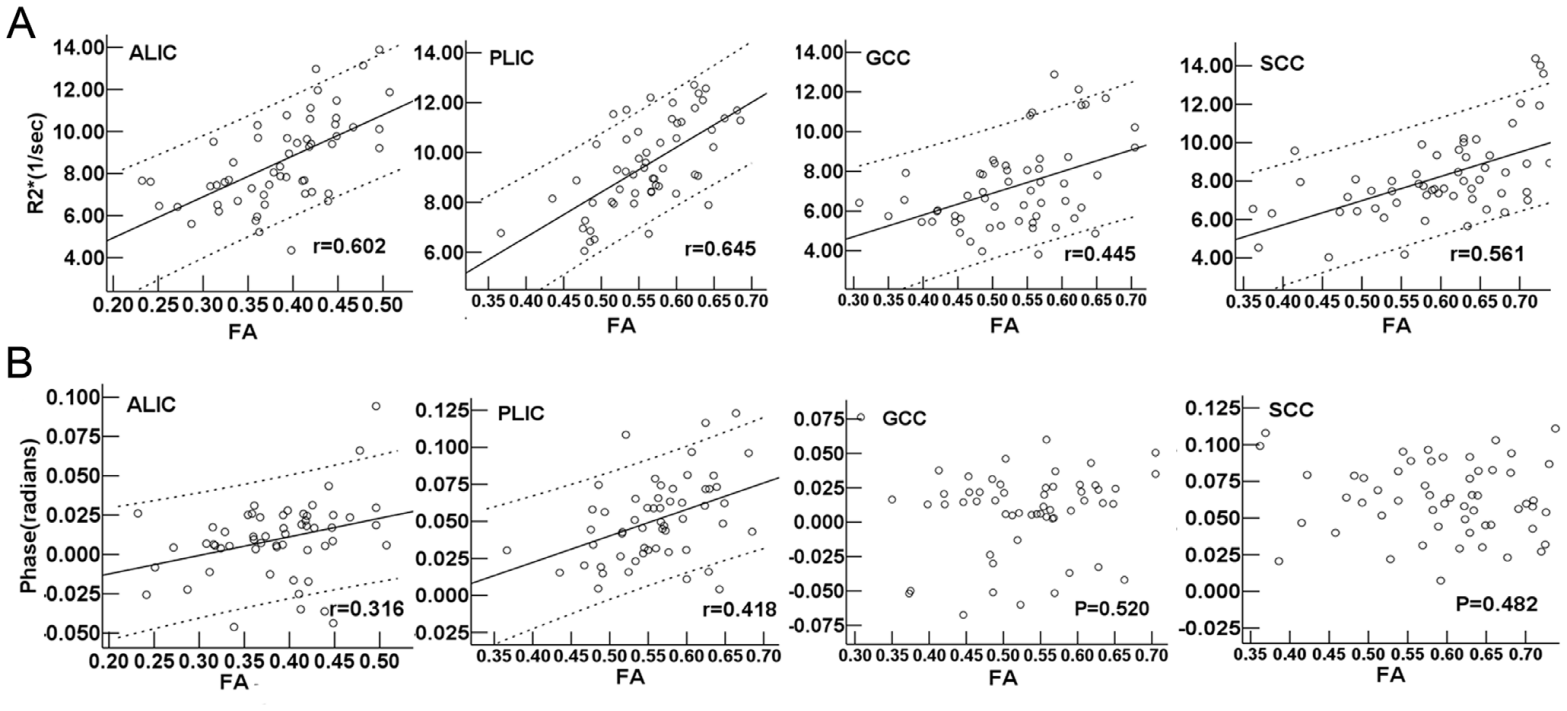
R2* and phase values vs. FA values in the white matter regions. (A) Upper: R2* vs. FA values, and (B) lower: phase vs. FA values. Pearson correlation analysis showed a positive correlation between the R2* values and FA values in the white matter regions (*P*<0.01), and between phase values and FA values in ALIC and PLIC (*P*<0.05). r is the coefficient of correlation. As for phase values, no correlations with FA values in GCC and SCC were found (*P*>0.05). FA: fractional anisotropy; ALIC: anterior limb of the internal capsule; PLIC: posterior limb of the internal capsule; GCC: genu of the corpus callosum; SCC: splenium of the corpus callosum.

FA values of the ALIC, PLIC, GCC and SCC all showed moderate positive correlations with PMA (r = 0.656, 0.648, 0.479 and 0.589 respectively, *P*<0.001). Moreover, moderate positive correlations were found between the R2* and FA values in the ALIC, PLIC, GCC and SCC (r = 0.602, 0.645, 0.445 and 0.561, respectively, *P*<0.01, Figure 8A). The poorly positive correlations between the phase and FA values were found just in the ALIC and PLIC (r = 0.316 and 0.418, respectively, *P*<0.05), and no significant correlations between the phase and FA values were found in the GCC and SCC (*P*>0.05) (Figure 8B).

In order to observe and compare the variation of the iron content and WM maturation in certain weeks of age by R2* values, the subjects were divided into eight groups according to the PMA [25] (Table 1 and 2). In neonates, the R2* value of the CN was the lowest among the six deep gray nuclei (P<0.01), and those of the RN and SN were much higher than other regions (P<0.05). The R2* values in the PLIC and SCC were higher than those in the ALIC and GCC (P = 0.047 and 0.041, respectively). Close to 1-year-old, the R2* values had no significant differences among all the deep gray nuclei (P>0.05) and anteroposterior differences between the WM regions (P>0.05).

**Table 1:** The R2* values (1/sec) in the six deep gray nuclei in eight periods (Mean ± SD, n = 56).

group	M/n	CN	PUT	GP	THA	RN	SN
37≤PMA<41	2/6	6.29±0.58	7.46±0.80	7.81±0.63	7.64±0.80	8.53±2.63	9.16±1.67
41≤PMA<46	7/13	6.95±1.24	8.26±1.07	7.55±1.44	7.87±1.00	10.10±1.51	10.07±1.35
46≤PMA<51	8/11	7.19±0.74	8.83±0.87	8.43±0.98	8.96±0.77	10.50±1.50	9.50±1.16
51≤PMA<56	4/6	8.08±1.06	8.38±1.11	8.31±1.02	9.08±1.28	10.31±1.21	9.60±0.79
56≤PMA<61	4/5	8.55±1.71	8.89±1.31	8.83±1.40	9.78±0.88	10.60±1.30	10.54±1.63
61≤PMA<66	5/6	7.45±1.27	9.38±1.46	9.08±1.45	9.88±0.95	11.23±2.21	10.23±1.18
66≤PMA<76	2/3	9.38±0.43	10.44±0.32	10.03±0.53	10.14±0.68	10.84±0.60	11.46±1.34
76≤PMA<91	4/6	10.58±0.60	10.81±0.19	10.98±0.84	11.00±0.33	11.06±0.88	11.43±0.86

Note: Infants were divided into eight groups according to the postmenstrual age (PMA).

M: male; CN: caudate nucleus; PUT: putamen; GP: globus pallidus, THA: thalamus; RN: red nucleus; SN: substantia nigra.

**Table 2:** The R2* values (1/sec) in the four WM regions in eight periods (Mean ± SD, n = 56).

group	M/n	ALIC	PLIC	GCC	SCC
37≤PMA<41	2/6	6.70±0.51	7.78±0.80	6.16±0.91	7.11±064
41≤PMA<46	7/13	7.08±1.04	7.89±1.28	5.38±1.10	7.06±1.97
46≤PMA<51	8/11	8.23±1.23	9.18±0.90	6.59±1.03	7.43±1.43
51≤PMA<56	4/6	8.51±1.01	9.84±1.07	6.44±1.20	7.60±0.71
56≤PMA<61	4/5	9.34±2.80	10.47±1.50	7.86±0.82	7.65±0.62
61≤PMA<66	5/6	8.66±1.74	10.67±1.36	8.24±1.72	9.16±0.97
66≤PMA<76	2/3	10.90±0.70	12.03±022	9.83±1.77	9.57±2.33
76≤PMA<91	4/6	12.34±1.30	11.86±0.62	11.26±1.43	12.31±2.10

Note: Infants were divided into eight groups according to the postmenstrual age (PMA).

M: male; ALIC: anterior limb of the internal capsule; PLIC: posterior limb of the internal capsule; GCC: genu of the corpus callosum; SCC: splenium of the corpus callosum.

## Discussion

To our knowledge, this is the first study to compare the properties of phase and R2* for assessing brain maturation in infant brains. The sequence named ESWAN was used to assess the age-related changes of the local R2* and phase values in the deep gray nuclei and WM regions. The results indicated that R2* is more sensitive than phase values to variations in PMA, brain iron concentration and FA. Therefore, it provided further support to select R2* as a sensitive marker for iron deposition and WM maturation in the brain during the infancy.

### Variation of R2* and Phase in Deep Gray Nuclei

Postmortem studies have demonstrated that iron levels in deep gray regions increased with age in normal individuals [11], [23], [26]. Non-heme brain iron residing in ferritin and hemosiderin molecules with a sufficient concentration could affect MR contrast [27] and lead to the increase of R2* [18], [28]. In a recent autopsy study [11], R2* was demonstrated as a preferred parameter for assessing iron concentration. In this study, we found a strongly positive correlation between the R2* and the iron concentrations, which indicated R2* may reflect the iron depositon in infants brain. Moreover, the changes of R2* also documented that the brain iron deposition increased gradually with age during the infancy. In addition, these findings of age-related changes in R2* were partly supported by previous studies, in which Ling et al. [19] illustrated a positive tendency between R2* values and PMA in neonates, and the age-related difference of T2* in brains between adults and newborns suggested a pattern of T2* reduction was developmentally dependent [29]. We found that in neonates, the R2* values in the RN and SN were higher than those in other deep gray nuclei, which were in accordance with previously reported studies in adults [1], [10], [30]. However, the R2* value in the CN was the lowest on birth, and up to 1-year-old, the R2* values were similar in all of the deep gray nuclei, which indicated that deep gray nuclei accumulated ferritin at different rates during different ages [23] and the iron deposition in the CN was most rapid in the first year of life.

The ability of phase value to reflect brain iron deposition remains controversial. Some studies reported that the phase value could reflect the increase of brain iron deposition in adults [7], [18], however, others showed that [5], [28] the phase value was less sensitive than FDRI or R2* to detect iron concentrations in brain regions. In this study, we found that there was no correlation between phase values and PMA or iron content in infant’s brain. As a parameter that reflects iron variation in the brain, phase value depends highly on filtering, structure size, shape and local environment. The edge effect could cause the fluctuation of phase values [31] and the difference of the magnetic susceptibility in the surrounding tissue besides the iron content [28], which would reduce the apparent phase shift in large uniform structures such as deep gray nuclei [28], [32]. Furthermore, blood volume as well as higher ferritin content both contributed to the contrast of gray matter and WM in phase imaging [33], and the phase shift also depended on the developmental variation of the cerebral venous system in infants [34]. In general, the phase imaging is more sensitive to some physical factors, such as main-field inhomogeneities, flowing or moving spins, the ratio of oxy- and deoxy-hemoglobin [5], neighboring susceptibility sources [35] and the type of background phase removal method [15], [35], [36]. Therefore, phase value may not be a suitable tool for quantifying brain iron accumulation during infancy.

### Variation of R2* and Phase in WM

Previous studies indicated that FA has the ability to reflect the complicated information of WM development including water content, axonal growth and myelination [37]–[39]. In this study we showed that R2* values in the deep WM increased with PMA and FA, indicating that R2* could reflect WM maturation in infants. The increased R2* value we observed may be due to several reasons. Firstly, the myelination is considered to be a primary source for the variation of the R2* [8]. During the infancy, a rapid change of water pool fractions in WM has been indicated in a three-pool relaxation model [40]. The increased bound water fractions of myelin and myelinated-axon both caused an increase of the R2* values [12]. Secondly, according to Todorich et al. [5], iron is an essential trophic factor that influences the production and maintenance of normal myelin, and the accumulation of iron by developing oligodendrocytes is an important event in the developmental brain. Thus, iron accumulation serves as a likely contributor to the increased R2* values.

It has been proved that myelin was an important and dynamic source of phase contrast [41], and the contributions from myelin in phase value also showed an age-dependent change [21]. In our study, the phase value in the internal capsule was found to increase with PMA as well as the FA value, indicating the rapid maturation in the first year of life [38], [42]. However, the phase values in the corpus callosum showed no correlation with PMA and FA. Tissue composition and architecture could be both responsible for this phase variation. In addition to speculative considerations, it was recently shown in vivo that phase shifts may be also influenced by the orientation of the underlying WM fibers with respect to the main magnetic field [43], [44]. Based on phase images, the dependency of gradient echo frequency shift on the orientation in highly myelinated human corpus callosum tissue specimens indicated that the microstructural orientation also presumably affected the variation of phase values in our study [45], which may also partly influence the age-related changes in the internal capsule. The infant’s head couldn’t rotate and modulate the direction like adults, so it’s difficult to obtain the complete and objective phase information according to the multiple orientations.

In neonates, the higher R2* and phase values in posterior WM indicated the PLIC and SCC developed earlier than ALIC and GCC respectively, which was consistent with the regular pattern of backward to forward WM maturation [46], [47]. Close to 1-year-old, the R2* values showed no significant anteroposterior differences, which indicated a rapid development of ALIC and GCC during the first year of life.

### The Limitations of this Study

Our study contains some limitations. Firstly, the brain regions we chose would change by age and reflect the progress of iron deposition and WM maturation [23], which were considered as the representative structures for brain development in previous studies [1], [38]. Whole brain research may avoid arbitrary in selecting ROIs and be more objective. However, we couldn’t find appropriate templates for phase and R2* images. Because of the higher brain water content and the much lower iron concentration in infants, the boundaries of many brain structures are not clear enough for automatic segmentation and registering. Secondly, brain water in infants was more than that in adults, and it generally decreased by age. The reduced water fractions may have influenced the change of the R2* value by age [48], [49], so we would choose more specific methods such as FDRI, R2’ or quantitative susceptibility mapping (QSM) [50] for further study. Furthermore, considering the potential influence on R2* and phase values by sedation, some correction using systemic oxygenation factors such as pressure of oxygen or carbon dioxide and oxygen saturation should be performed in different individuals [34]. Further research would also need to focus on the improvements of appropriate filter strengths, structure measurement areas [31], and methods of calculation [13], [19] in order to make the phase value more reliable and objective.

In conclusion, we compared the R2* and phase values to detect the variations in PMA, brain iron concentration and FA in brain during the first year of life, and found that R2* is a preferable MR parameter for in vivo estimation of iron content and WM maturation in early brain development, while phase value has limitations.
